# Morphological, cytochemical and ultrastructural aspects of blood cells in freshwater stingray species in the middle Rio Negro basin of Amazonian Brazil

**DOI:** 10.1038/s41598-021-95183-4

**Published:** 2021-08-03

**Authors:** Adriano Teixeira de Oliveira, Jefferson Raphael Gonzaga de Lemos, Marcio Quara de Carvalho Santos, Jackson Pantoja-Lima, Paulo Henrique Rocha Aride, Maria Lúcia Góes de Araújo, Marcos Tavares-Dias, Jaydione Luiz Marcon

**Affiliations:** 1 Instituto Federal de Educação, Ciência e Tecnologia do Amazonas (IFAM), Campus Manaus Centro (CMC), Avenida Sete de Setembro, 1975. Centro, Manaus, AM 69020-120 Brazil; 2 Faculdade Estácio do Amazonas, Avenida Constantino Nery, 3693. Chapada, Manaus, AM 69050-001 Brazil; 3 Programa de Pós-Graduação em Ciências Pesqueiras nos Trópicos (PPG-CIPET), Universidade Federal do Amazonas (UFAM), Avenida General Rodrigo Octávio Jordão Ramos, 3000. Coroado I, Manaus, AM 69077-000 Brazil; 4 Instituto Federal de Educação, Ciência e Tecnologia do Amazonas (IFAM), Campus Presidente Figueiredo, Avenida Onça Pintada, 1308. Galo da Serra, Presidente Figueiredo, AM 69735-000 Brazil; 5 Universidade Federal de Sergipe (UFS), Avenida Marechal Rondon, sn. Jardim Rosa Elze, São Cristovão, SE 49100-000 Brazil; 6 Empresa Brasileira de Pesquisas Agropecuárias (EMBRAPA), Macapá, Rodovia Juscelino Kubitschek km 5, Macapá, AP 68903-419 Brazil; 7 Laboratório de Ciências Fisiológicas, Universidade Federal do Amazonas (UFAM), Avenida General Rodrigo Octávio Jordão Ramos, 3000. Coroado I, Manaus, AM 69077-000 Brazil

**Keywords:** Cell biology, Physiology

## Abstract

In the present work, we examined the morphology, dimensions, cytochemical staining reactions and ultrastructure of blood cells from three freshwater stingray species, *Potamotrygon wallacei, Potamotrygon motoro* and *Paratrygon aiereba,* living in the waters of the middle Rio Negro basin (Barcelos, Amazonas, Brazil). We identified erythrocytes, erythroblasts, thrombocytes and four types of leukocytes (basophils, heterophils, lymphocytes and monocytes) in the blood of these stingray species. In all the freshwater stingray species studied, the shapes and dimensions of these cells were similar to those of marine elasmobranchs. Positive PAS staining occurred in heterophils and thrombocytes, and weak staining occurred in lymphocytes and monocytes, while metachromasia only occurred in basophils. Positive Sudan Black B staining was observed in thrombocytes and lymphocytes, and weak staining occurred in heterophils. Basophils and heterophils were the only cells with positive bromophenol blue staining, while no peroxidase staining was observed in any of the four leukocyte types. This is the first study to establish the dimensions and cytochemical staining profiles of blood cells in Amazonian stingray species. Because these elasmobranch species are exported as ornamental fish to countries worldwide, this study can contribute to establishing standards for blood constituents that may be helpful in assessing the health and welfare of these fish in artificial systems.

## Introduction

The subfamily Potamotrygoninae is a unique elasmobranch group composed of freshwater stingray species distributed along most of the great fluvial systems of South America ending at the Atlantic Ocean or Caribbean Sea^1–3^. There are four genera of freshwater stingrays: *Plesiotrygon*, *Paratrygon*, *Potamotrygon*^4^ and *Heliotrygon*^5^. However, great effort and research investment are needed to achieve a better understanding of the diversity and taxonomic status of this family^6^.

Freshwater stingrays are an important component of Amazonian biodiversity. They have great socioeconomic importance, especially because of their use in the international ornamental fish trade and because they represent an alternative source of income for riverine communities living along the tributaries of the middle Rio Negro basin^7^. There is a relationship between freshwater stingrays and fishermen, especially because stingray stingers can cause accidents^8,9^. Four valid stingray species are found in the black waters of the Rio Negro basin: *Potamotrygon motoro* (Müller & Henle 1841)*, P. orbignyi* (Castelnau 1855)*, P. schroederi* (Fernández-Yépez 1958) and *Paratrygon aiereba* (Müller & Henle 1841). In addition, a new species known as *P. wallacei* (cururu stingray) (Carvalho et al. 2016) is currently being identified and scientifically characterized. This species is probably endemic to this region, with a hotspot concentrated in the Mariuá archipelago near the municipality of Barcelos (Amazonas, Brazil)^10^.

Investigations on the blood constituents of several marine elasmobranch fish species have been conducted, especially in sharks^11–16^. Nevertheless, only a few studies have addressed freshwater elasmobranchs^15,17–23^. The presence of erythrocytes, thrombocytes, lymphocytes, monocytes, heterophils and basophils in freshwater stingrays of the Potamotrygonidae family has been reported^21^. However, these authors did not investigate the cytochemical features of these cell types to confirm their identities by examining traditional morphology from cytochemical markings.

Hematological evaluations are becoming routine practice for assessing the health of fish and other animals^19,21,24–33^. Studies on blood leukocytes can reveal the characteristics of the immune systems of different fish species^34,35^, including free-living Amazonian stingrays. Hematological investigations have relied on classical Romanowsky staining with the Leishman, Wright, May, Grünwald and Giemsa used to identify leukocytes^36–38^, but cell-based classifications of stingray leukocyte cells are not always reliable using classical staining methods because the staining procedures vary, which can lead to errors in the identification of a cell type. Thus, cytochemical staining of leukocytes in blood may be particularly useful for identifying cell lineages and may indicate cell function.

This study aimed to investigate the morphology, dimensions, cytochemical staining reactivity and ultrastructure of blood cells from three freshwater stingray species, *P. wallacei* (cururu stingray), *P. motoro* and *P. aiereba*, living in the black waters of the middle Rio Negro basin (Barcelos, Amazonas, Brazil). Because Brazil and other Amazonian countries export these species as ornamental fish to consumers around the world, these results will contribute to establishing standards for blood constituents that may be helpful in assessing the health and welfare of these fish in artificial systems, especially in relation to the ornamental fish trade.

## Materials and methods

### Study area and specimen collection

Specimens of the Amazonian stingrays *Potamotrygon wallacei* (cururu stingray; n = 53), *Potamotrygon motoro* (n = 55) and *Paratrygon aiereba* (n = 32) were collected from the Mariuá archipelago (license: 15116-1 IBAMA). This is the largest complex of islands that exists in continental waters (more than 700 islands), and it is located in the black waters of the middle Rio Negro basin near the municipality of Barcelos (Amazonas, Brazil). The fish were caught at different sites within the archipelago, including beaches, lakes, small streams (igarapés), and areas of flooded forest (igapós), between January 2006 and October 2019. Professional fisherman caught the fish at night (19:00 to 03:00) through active searching with the aid of a head flashlight, paddle and typical hand net (rapiché). We immediately anesthetized the captured stingrays with eugenol (0.2 g L^−1^) and withdrew a blood sample (1.0–1.5 mL) from the gill arterial vessel^18^ using 10% EDTA as an anticoagulant^20^. After these procedures, we measured the total length (TL, cm), disc width (DW, cm) and body weight (BW, kg) of each specimen. All the stingrays sampled recovered from the anesthetic and were safely returned to their respective capture sites.

For cytochemical staining and ultrastructural examination of different blood cell types, ten *P. wallacei, P. motoro* and *P. aiereba* stingrays were caught by professional fishermen near the Daracuá community within the Mariuá archipelago. These stingrays were transported by boat (journey of 24 h) to the Laboratory for Physiology Applied to Aquaculture (LAFAP) at the National Amazon Research Institute (Instituto Nacional de Pesquisas da Amazônia, INPA) in Manaus (Amazonas, Brazil). At the laboratory, the stingrays were acclimatized in 5000-L tanks for 48 h, with constant water changes and oxygenation so that they would recover from the stress of being captured and transported. After this period, a blood sample (1.0 mL) was collected from the gill arterial vessel using 10% EDTA as an anticoagulant^20^. Then, the biometric parameters were determined (TL, DW and BW).

### Morphological and morphometric measurements and quantification of blood cells

Fresh blood samples were collected from *P. wallacei* (n = 43), *P. motoro* (n = 45) and *P. aiereba* (n = 32). We stained these blood smears with a combination of May-Grünwald-Giemsa-Wright stains to identify cells and make morphometric measurements (µm) of 100 samples of each cell type found (erythrocytes, leukocytes and thrombocytes), with the aid of an optical microscope and a millimeter ruler for determination of the largest and smallest cells.

Subsequently, the blood samples were used for leukocyte and total thrombocyte counts^37^ and for differential leukocyte counts, which were based on the counts of 200 leukocytes^21^.

### Cytochemical staining

We collected fresh blood samples from 10 specimens of each stingray species. The presence and intensity of glycogen deposits inside blood cells was confirmed by using the periodic acid-Schiff (PAS) method. Controls for this reaction were obtained through smears exposed to salivary amylase digestion for 60 min.

The peroxidase reaction was carried out by using the ortho-toluidine method in the presence or absence of hydrogen peroxide. The reaction products were subjected to nuclear staining using Harris hematoxylin^37^.

Reactions for metachromasia were tested in blood smears fixed in 1% lead subacetate for 10 min and subsequently stained with 0.2% toluidine blue for 50 min^37^. The presence of lipids in different blood cell types was confirmed in blood smears previously fixed with 70% ethanol for 5 s and then stained with 0.3% Sudan Black B solution^26^.

To identify total protein, blood smears were fixed in formalin, stained with bromophenol blue for 15 min, immersed in 0.5% acetic acid, washed in phosphate buffer and finally dehydrated in butyl alcohol. Reticulocytes were identified using a solution of brilliant cresyl blue and blood (1:1), which was homogenized, kept in a water bath for 20 min at 37 °C and stained with a combination of May-Grünwald-Giemsa-Wright stains^37^. The results from the cytochemical staining were expressed qualitatively, according to the intensity of staining observed for each blood cell type, i.e., negative staining (–), weakly positive staining (+) and positive staining (++).

### Ultrastructural analysis

The blood cell types were characterized ultrastructurally in four of the ten stingrays from each species that had been acclimatized for cytochemical studies. Blood samples were taken from the gill vessel^18^ and centrifuged at 750*g* for 15 min to obtain pellets containing erythrocytes, thrombocytes and leukocytes. We immediately fixed these pellets in 0.1 M sodium cacodylate solution (pH 7.4) containing 2.5% glutaraldehyde and 2.0% paraformaldehyde at 4 °C for 2.5 h. We then immersed these samples in a 0.2 M sodium cacodylate solution (pH 7.4) containing 1% osmium tetroxide at 4 °C for one hour. After these procedures, the samples were dehydrated and embedded in Araldite resin (Sigma-Aldrich, USA), and sections were cut using a Reichert OM-U3 ultratome, mounted on copper grids (200 mesh) and stained with 0.2% uranyl acetate solution and lead citrate solution for 15 min. The sections were analyzed using a transmission electron microscope at the Microscopy Center of the Institute of Biosciences at São Paulo State University (Universidade Estadual Paulista Julio de Mesquita Filho, UNESP) in Botucatu of São Paulo, Brazil.

### Ethical approval

The study was conducted at the Department of Physiological Sciences, Federal University Amazonas, in accordance with the Brazilian guidelines for animal experiments and was approved by the government of Amazonas (license: 15116-1), Brazil. All experiments were conducted according to local and ARRIVE guidelines^39^.

### Research involving human participants and/or animals

A total of 140 rays were captured in the natural environment, 130 of which were returned, and 10 were processed and registered in the collection.

## Results

The mean values for total length, disc width and body mass of *P. wallacei*, *P. aiereba* and *P. motoro* specimens are shown in Table 1.

**Table 1:** Means (cm) ± standard deviations of the biometric variables for *P. wallacei*, *P. motoro* and *P. aiereba* in the Middle Rio Negro of the Amazon are shown.

Species	Total length (cm)	Disk width (cm)	Body weight (g)
*P. wallacei*	19.1 ± 2.5	17.4 ± 1.1	226.0 ± 48.5
*P. motoro*	25.1 ± 3.1	20.4 ± 1.8	351.0 ± 65.0
*P. aiereba*	44.8 ± 13.9	29.3 ± 10.8	966.5 ± 856.9

### Morphological and morphometric measurements and quantification of blood cells

Blood smears from *P. wallacei, P. motoro* and *P. aiereba* revealed erythroblasts, mature erythrocytes, thrombocytes, lymphocytes, monocytes, heterophils and basophils of similar sizes among the species.

**Figure 1:**
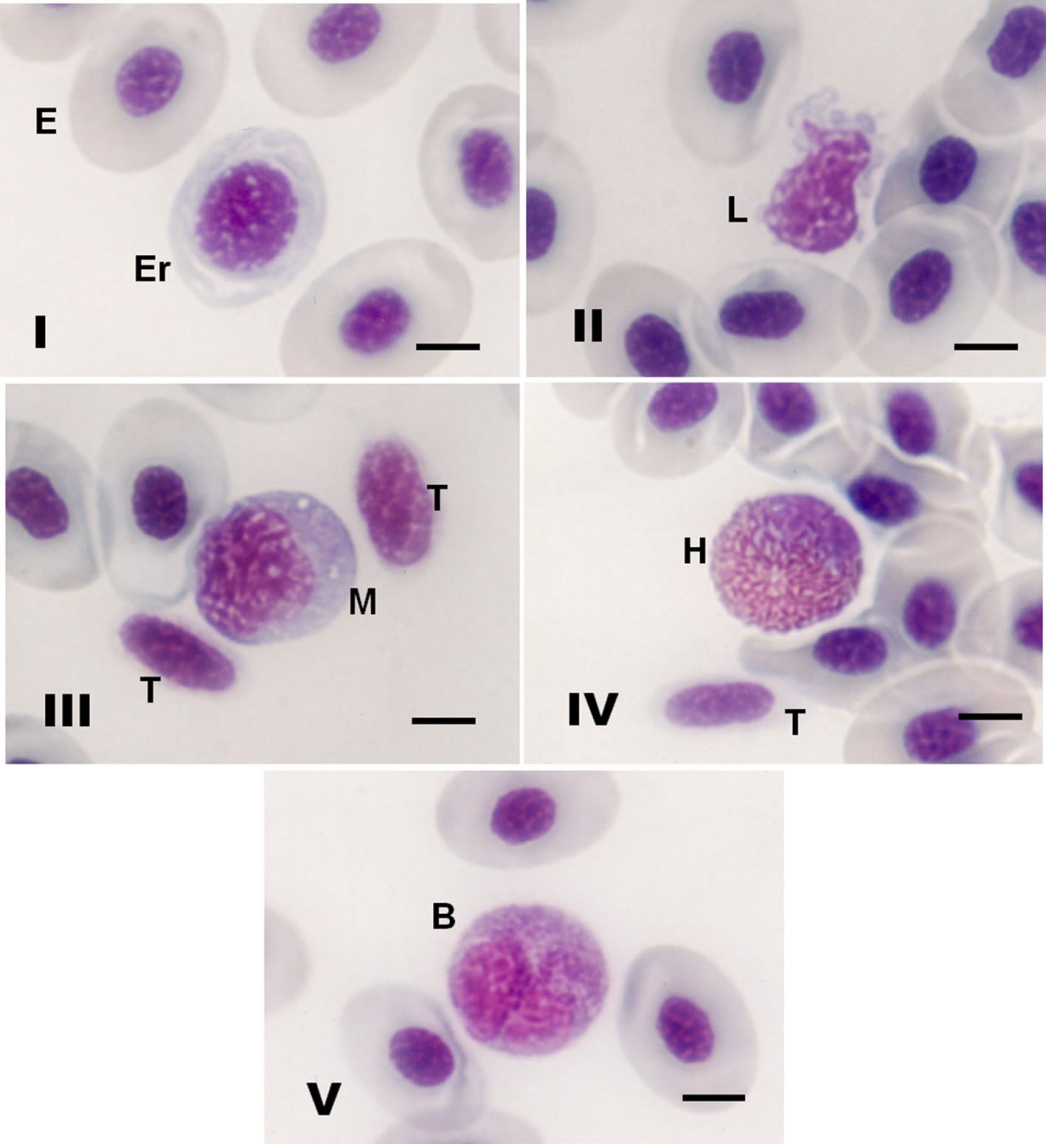
(**I**–**V**) Morphology of blood cells of three freshwater stingray species stained with May Grunwald-Giemsa-Wright stains. (**I**) (E) Erythrocytes and (Er) erythroblasts of *P. wallacei*; (**II**) (L) lymphocytes of *P. wallacei*; (**III**) (T) thrombocytes and (M) monocytes of *P. wallacei*; (**IV**) (H) heterophils and (T) thrombocytes of *P. wallacei*; (V) (B) basophils of *P. wallacei*. Bar = 8 µm.

Mature erythrocytes were very similar in shape and size in the three Amazonian stingray species. Under an optical microscope, mature erythrocytes presented as elliptical cells with abundant hyaline in the cytoplasm, and the nucleus was usually centered and condensed with a shape that resembled the cell shape (Fig. 1-I). Erythroblasts were rounded cells and were easily distinguished from mature erythrocytes by their pale appearance or by hyaline in the cytoplasm (Fig. 1-I).

Lymphocytes presented different sizes and irregular shapes, which were mostly elliptical and rarely oval, with a nucleus occupying a large part of the basophilic cytoplasm. Lymphocytes presented cytoplasmic projections without visible granulations and sometimes presented vacuoles (Fig. 1-II). Thrombocytes were generally fusiform, with hyaline in the cytoplasm; the nucleus occupied almost the entire cell and resembled the cell shape (Fig. 1-III). Monocytes were predominantly oval, with a nucleus morphology similar to that of thrombocytes (Fig. 1-III). Heterophils were predominantly oval, with a large amount of heterophilic coarse granules and a nucleus that was generally eccentric (Fig. 1-IV). Basophils were also predominantly oval, with basophilic granules and a nucleus that was eccentric and generally bilobulated (Fig. 1-V).

In comparison with the other leukocyte cell types, monocytes were the largest cells in the three elasmobranch species (Table 2). Basophils and lymphocytes were the smallest cell types in the blood of the three freshwater stingray species investigated herein (Table 2).

**Table 2:** Mean diameters (µm ± SD) of the largest and smallest axes of different blood cells (n = 50) from three freshwater stingray species living in the middle Rio Negro basin in Amazonas, Brazil are shown.

Cells	*P. wallacei*	*P. motoro*	*P. aiereba*
Erythrocytes (µm)	20.1 ± 0.7 × 14.1 ± 0.6	20.2 ± 0.8 × 14.1 ± 0.7	20.0 ± 0.8 × 14.0 ± 0.8
Erythroblasts (µm)	19.0 ± 0.9 × 14.8 ± 0.4	19.0 ± 0.8 × 14.7 ± 0.5	19.1 ± 0.7 × 14.8 ± 0.5
Thrombocytes (µm)	14.7 ± 1.4 × 9.6 ± 0.5	14.6 ± 1.5 × 9.5 ± 0.6	14.6 ± 1.3 × 9.6 ± 0.4
Lymphocytes (µm)	14.4 ± 1.8 × 12.4 ± 2.7	14.7 ± 1.7 × 12.8 ± 3.1	14.8 ± 2.1 × 12.7 ± 2.9
Monocytes (µm)	21.4 ± 1.1 × 21.4 ± 1.1	21.3 ± 1.2 × 21.3 ± 1.2	21.5 ± 1.0 × 21.5 ± 1.0
Heterophils (µm)	14.5 ± 0.5 × 14.5 ± 0.5	14.4 ± 0.4 × 14.4 ± 0.4	14.4 ± 0.5 × 14.4 ± 0.5
Basophils (µm)	13.5 ± 0.5 × 13.5 ± 0.5	13.4 ± 0.6 × 13.4 ± 0.6	13.6 ± 0.6 × 13.6 ± 0.6

Leukocyte and thrombocyte cell counts showed that lymphocytes and monocytes were the predominant blood cells, while heterophils and basophils were the least abundant blood cells in the three freshwater stingray species investigated (Table 3).

**Table 3:** Leukocyte and thrombocyte counts of *P. wallacei*, *P. motoro* and *P. aiereba* from the Middle Rio Negro of the Amazon are listed.

Species	*P. wallacei*	*P. motoro*	*P. aiereba*
Leukocytes (µL)	3629 ± 2001	2998 ± 1107	3297 ± 1469
Thrombocytes (µL)	890 ± 498	826 ± 601	690 ± 468
Lymphocytes (%)	46.1 ± 15.8	45.6 ± 10.9	43.6 ± 14.5
Lymphocytes (µL)	1673 ± 316	1367 ± 121	1437 ± 213
Monocytes (%)	30.7 ± 14.7	26.2 ± 4.3	28.5 ± 12.3
Monocytes (µL)	1114 ± 294	785 ± 51	939 ± 180
Heterophils (%)	20.2 ± 10.7	25.1 ± 15.5	24.9 ± 14.0
Heterophils (µL)	733 ± 214	752 ± 171	820 ± 205
Basophils (%)	3.0 ± 2.0	2.7 ± 0.6	3.0 ± 1.8
Basophils (µL)	109 ± 40	81 ± 7	99 ± 26

### Cytochemical staining

**Figure 2:**
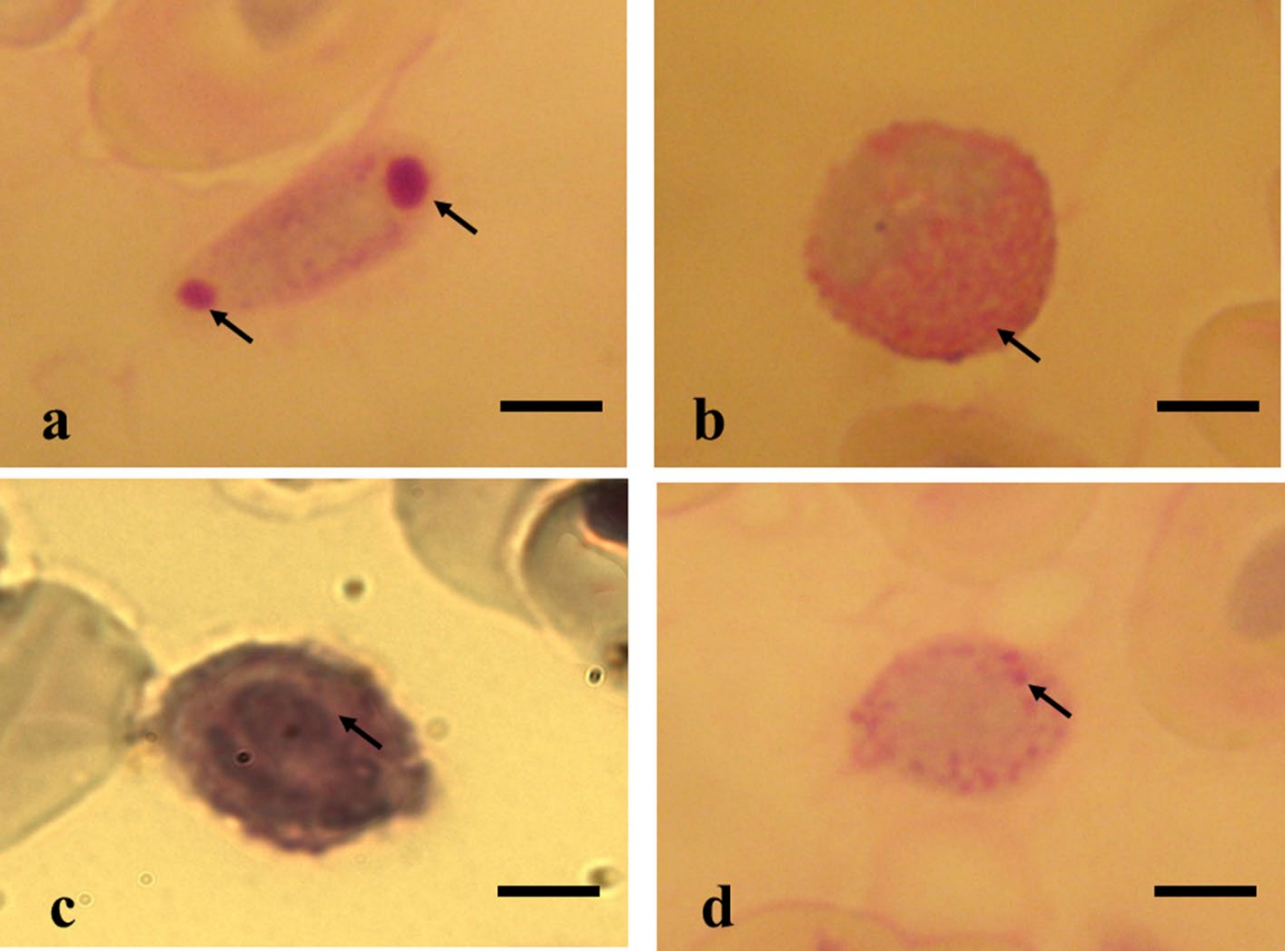
(**I**–**IV**) PAS staining for detection of glycogen in blood cells of freshwater stingrays in central Amazonia. (**I**) Thrombocytes of *P. aiereba*; (**II**) Heterophils of *P. aiereba;* (**III**) Lymphocytes of *P. wallacei;* (**IV**) Monocytes of *P. motoro*. Bar = 8 µm.

Thrombocytes and leukocytes did not show any differences in cytochemical staining when comparing between the three stingray species (Table 4). Glycogen marking was observed in thrombocytes (Fig. 2-I) and heterophils (Fig. 2-II), and there was weak positive staining in lymphocytes (Fig. 2-III) and monocytes (Fig. 2-IV).

**Table 4:** Cytochemical staining results of the blood cells of stingrays *P. wallacei*, *P. motoro* and *P. aiereba* from the middle Rio Negro of the Amazon are shown.

Cells	PAS			Peroxidase			Toluidine blue			Sudan Black B			Bromophenol blue		
	1	2	3	1	2	3	1	2	3	1	2	3	1	2	3
Thrombocytes	++	++	++	−	−	−	−	−	−	++	++	++	−	−	−
Lymphocytes	+	+	+	−	−	−	−	−	−	++	++	++	−	−	−
Monocytes	+	+	+	−	−	−	−	−	−	−	−	−	−	−	−
Heterophils	++	++	++	−	−	−	−	−	−	+	+	+	++	++	++
Basophils	−	−	−	−	−	−	++	++	++	−	−	−	++	++	++

(1) *P. wallacei*; (2) *P. motoro;* (3) *P. aiereba.*

− Negative; + weakly positive; ++ positive.

**Figure 3:**
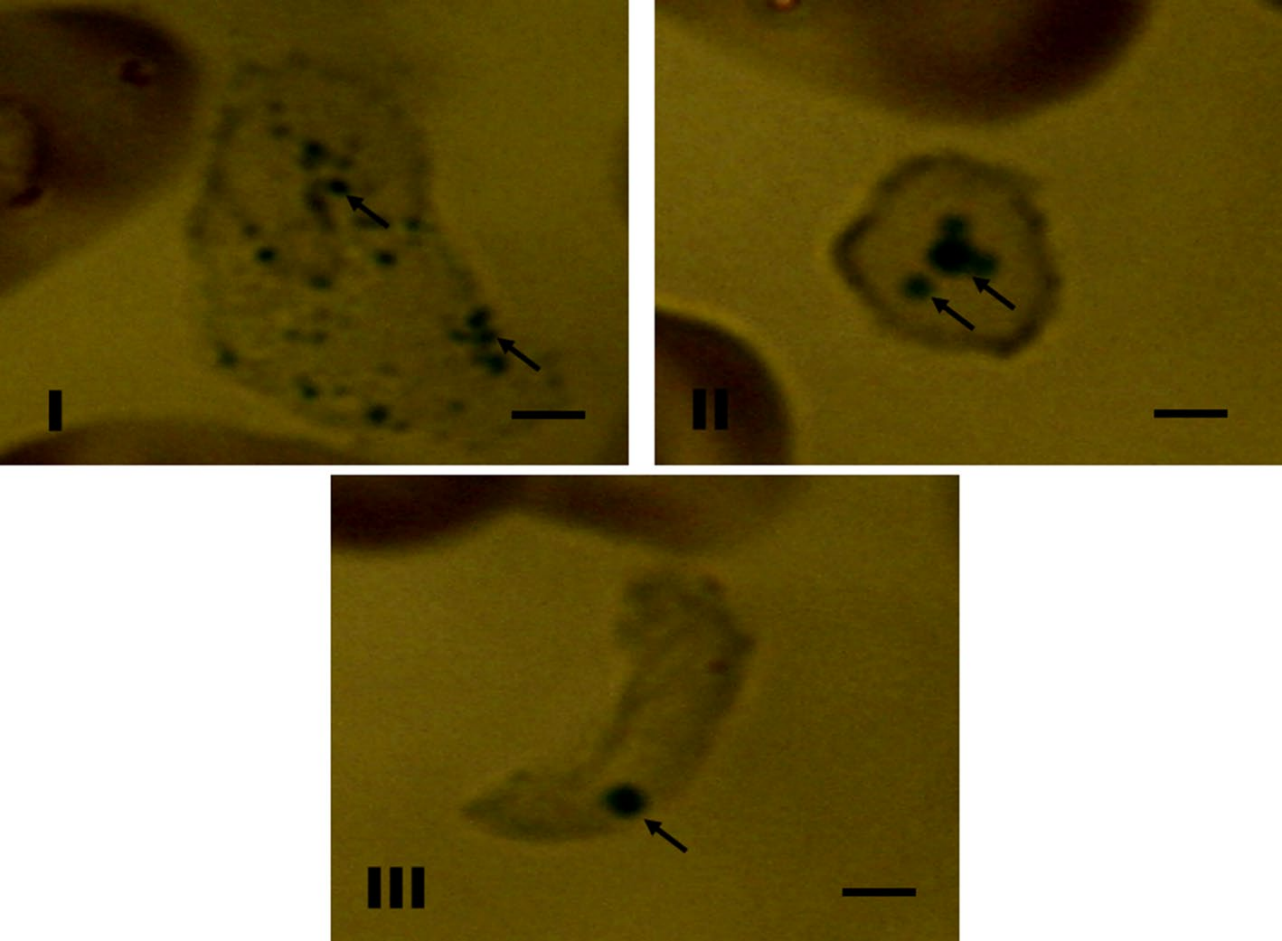
(**I**–**III**) Cytochemical staining of lipids with Sudan Black B was performed in blood cells of freshwater stingrays in central Amazonia. (**I**) Heterophils of *P. wallacei*; (**II**) Lymphocytes of *P. aiereba;* (**III**) Thrombocytes of *P. aiereba*. Bar = 8 µm.

**Figure 4:**
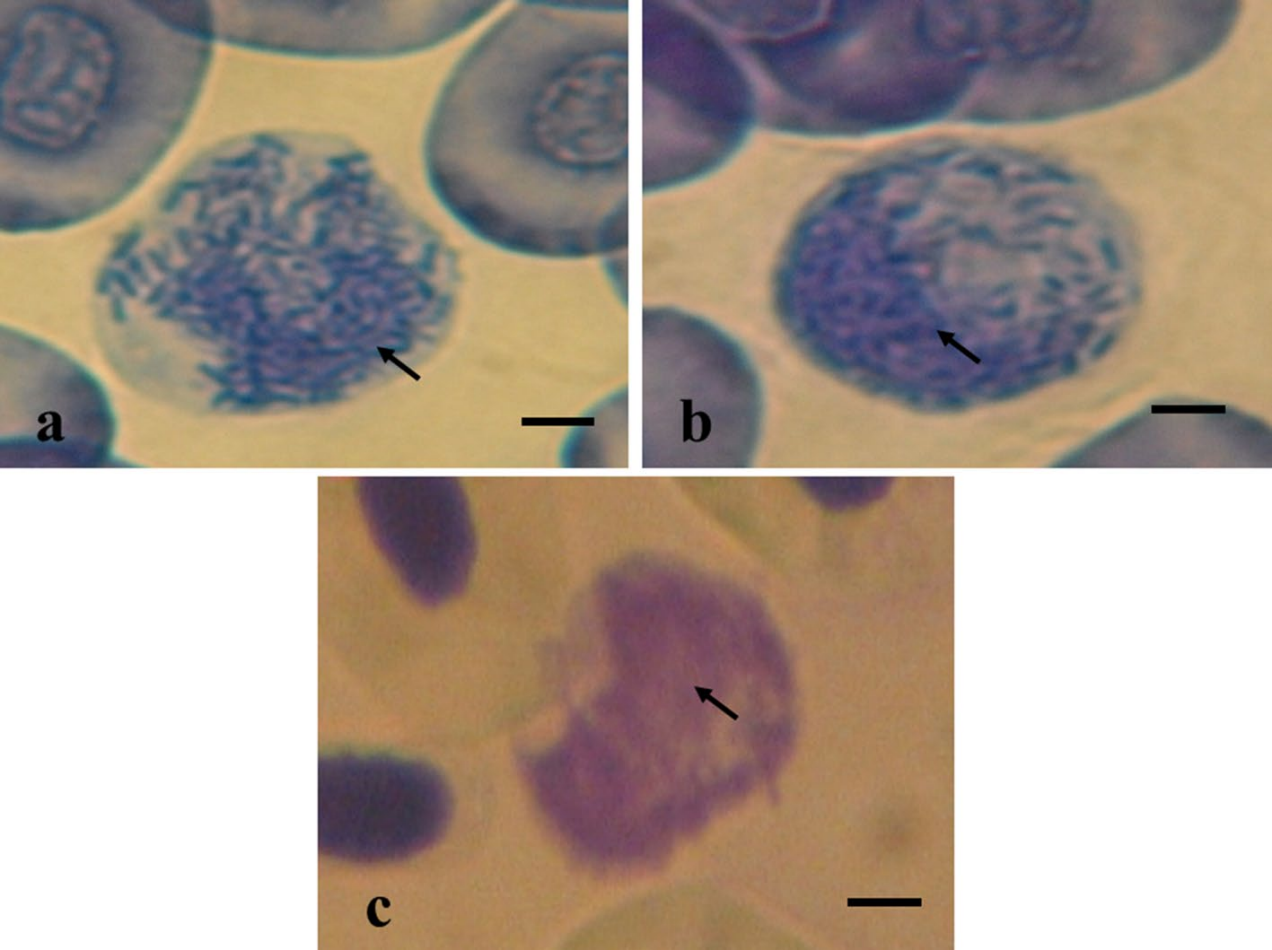
(**I**–**III**) Cytochemical staining of total protein and metachromasia in blood cells of freshwater stingrays in central Amazonia. (**I**) Total protein staining in heterophils of *P. motoro*; (**II**) Total protein staining in basophils of *P. wallacei*; (**III**) Metachromasia in basophils of *P. wallacei*. Bar = 10 µm.

Weak positive staining with Sudan black was observed in heterophils (Fig. 3-I), lymphocytes (Fig. 3-II) and thrombocytes (Fig. 3-III). Positive identification of proteins using bromophenol blue occurred only in granules of heterophils (Fig. 4-I) and basophils (Fig. 4-II). The presence of reticulocytes was observed in erythrocytes, thus indicating the presence of crosslinking material fragments that were not stained with traditional dyes. There was no positive peroxidase staining, although metachromasia was observed (Fig. 4-III). This was characterized using a blue reagent that reacted with the red-colored blood of the freshwater stingrays.

### Ultrastructural analysis

**Figure 5:**
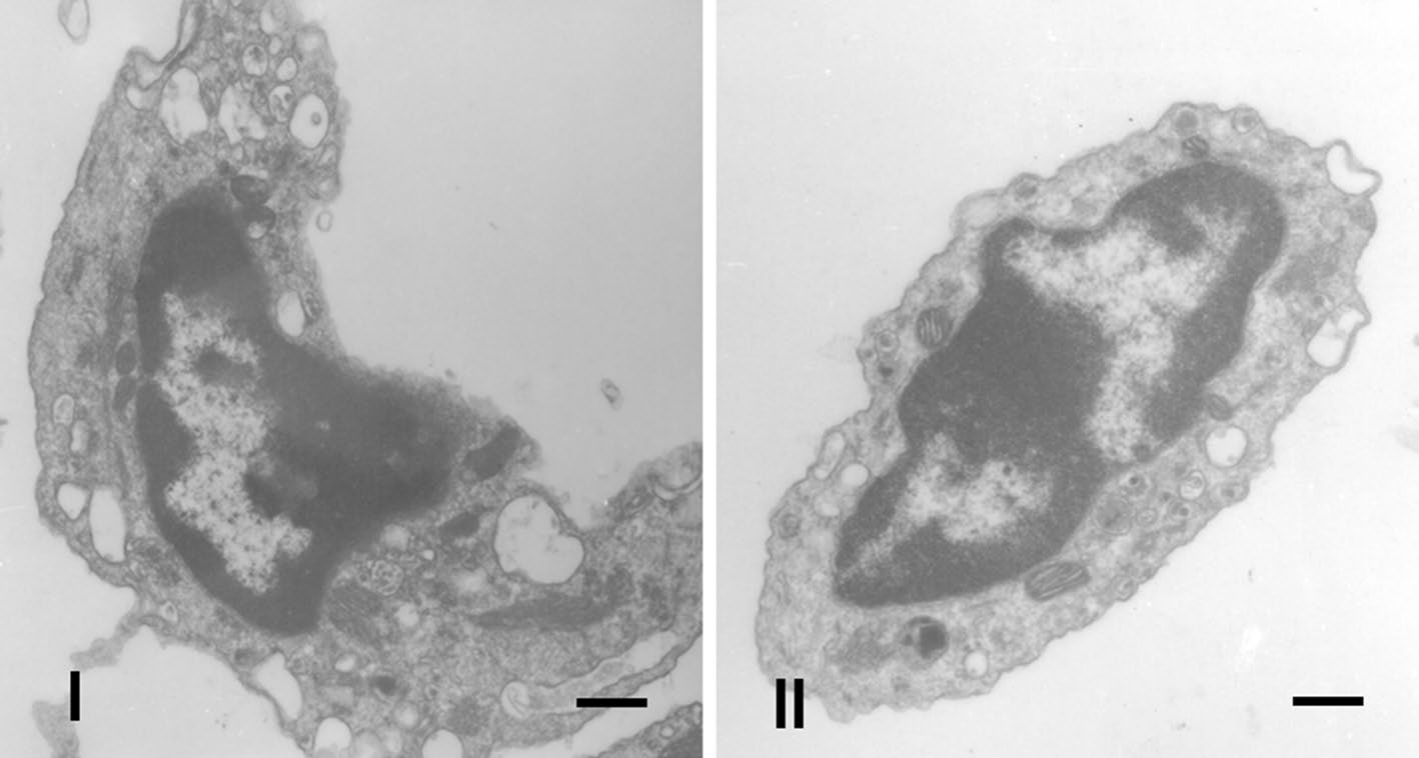
(**I**,**II**) Ultrastructural analysis of blood cells from freshwater stingrays in central Amazonia. (**I**) Thrombocytes of *P. motoro;* (**II**) Lymphocytes of *P. wallacei*. Increase 4000 x.

Thrombocytes were generally round and spindle-shaped. In the cytoplasm, a canalicular system with various-sized vesicles and canaliculi was occasionally observed, along with glycogen pellets, granules and numerous mitochondria (Fig. 5-I). Lymphocytes presented amorphous forms, with sparse cytoplasm. The presence of vacuoles and few mitochondria was observed, and the nucleus occupied almost the entire cell, with dense chromatin in the periphery and no evident nucleolus (Fig. 5-II). Monocytes presented nuclei with peripheral heterochromatin and cytoplasm with mitochondria, secretion vesicles, secretion granules and endoplasmic reticulum. Because basophils were scarce in the blood, they could not be found in the potamotrygonids in this study. Staining of heterophils revealed the presence of heterochromatin, and there were large numbers of granules that might have been glycogen, lipids and/or proteins, but they could not be distinguished.

## Discussion

### Morphological and morphometric measurements and quantification of blood cells

Most vertebrates have seven blood cell types: erythrocytes, thrombocytes, lymphocytes, eosinophils, basophils, monocytes and neutrophils^40,41^. The morphology of each cell type appears to be similar, except for neutrophils. In some cases, neutrophils are replaced by heterophils, which present the same immunological function^40,42,43^. It was reported that erythrocytes, thrombocytes, lymphocytes, monocytes, neutrophils and eosinophils were present in freshwater potamotrygonids^17^.

In contrast, no eosinophils were observed in blood from Amazon stingrays, thus suggesting that heterophils have some importance in the immune defense of these potamotrygonids. In addition, there is a lack of standardization in the staining procedures adopted and in the classification of blood cell types. In the present study, erythroblasts, mature erythrocytes, thrombocytes, lymphocytes, monocytes, heterophils and basophils were observed.

In the potamotrygonids of this study, reticulocytes were identified by the presence of ribonucleoproteins inside some erythrocytes. High amounts of ribonucleoproteins indicate premature release of erythrocytes into the bloodstream^37^. Therefore, quantification of the number of circulating reticulocytes can provide information about erythropoietic activity and therefore about the animal health status.

The morphological features of freshwater stingray erythrocytes are similar to those of marine elasmobranchs, such as the *Dasyatis sabina* (Lesueur 1824), *Raja eglanteria* (Bosc 1800)^13,16^, *Raja microocellata* (Montagu 1818), *R. brachyura* (Lafont 1871) and *Raja* sp.^44^ stingrays and the *Squalus acanthias* (Linnaeus 1758)^45^, *Schroederichthyes chilensis* (Guichenot 1848)^12^, *Ginglymostoma cirratum* (Bonnaterre 1788) and *Carcharhinus limbatus* sharks (Müller & Henle 1839).

In addition, in the *C. coelolepis* shark, immature erythrocytes (erythroblasts) may be smaller than mature erythrocytes^46^, and this characteristic was also observed in the three potamotrygonid stingray species.

Thrombocytes in elasmobranchs are blood cells with functions analogous to mammalian platelets, which play a role in homeostasis^13,16^. In dogfish (*S. canicular*), it was demonstrated that blood thrombocytes remove antigenic substances, such as colloidal charcoal particles^47^. The cell sizes and morphological characteristics of freshwater stingray thrombocytes were similar to those reported in the *S. chilensis*^12^ and *C. leucas*^13^ sharks and different from those of *C. plumbeus,* which presented cytoplasmic granules^11^. Moreover, in the blood of the *C. coelolepis* shark, the form known as "drop" (with fingerlike cytoplasmic projections) was observed^46^, but this was not detected in the Amazonian stingrays herein.

In blood smears from marine elasmobranchs, leukocytes at different stages of maturation are frequently observed. This can cause incorrect identification^13^, thereby contributing to the confusing terminology of elasmobranch leukocytes^13^ and causing errors in identifying small monocytes and large lymphocytes^48^. In the present study, lymphocytes presented shapes ranging from round to amorphous, and this has also been observed among lymphocytes in *C. coelolepis*^46^, *S. chilensis*^12^, *G. cirratum*^12,13^, *C. plumbeus*^11^, *R. microocellata, R. brachyura, R.* sp.^44^, *O. maculatus, O. ornatus, Orectolobus* sp.^49^ and *R. typus*^15^. The size of the lymphocytes in the three Amazonian stingray species was slightly smaller than that of the *C. coelolepis* shark^46^.

The lymphocyte morphological characteristics were similar to those observed in other elasmobranchs^11–13,15,44,49^. Granulocytes have been reported in several elasmobranch species, but they are difficult to identify and classify because of the great variations in shape and size and the poor staining of the cells^12^. In the present study, in the blood of freshwater stingrays, two types of granulocytes were detected: heterophils and basophils. It was reported that the most common granulocytes in the blood of elasmobranchs were heterophils, while basophils were rare in blood^12^. It was reported that neutrophils and eosinophils were present in the blood of potamotrygonids^17^. The identification of neutrophils and eosinophils in potamotrygonids can be correlated with the extreme difficulty of the methods for staining smears and/or with incorrect classification of the different types of leukocytes. Heterophils and basophils with the same morphological features observed in the three Amazonian stingrays were also observed in *C. coelolepis* (dogfish shark)^46^, *S. chilensis* (catshark)^12^, *C. limbatus* (blacktip shark)^13^ and *R. typus* (whale shark)^15^.

Erythrocytes are generally larger in lower orders, and variations in size may occur within species of the same order^40^. The freshwater stingray erythrocytes were smaller than those of the *Centroscymnus coelolepis* shark (Barbosa du Bocage & de Brito Capello 1864)^46^ and approximately two times larger than those in freshwater and marine teleosts^50^ and in the *Dicentrarchus labrax* L. fish^51^.

The number of total leukocytes and thrombocytes observed in the present study was similar to the number reported for freshwater stingrays *P. falkneri, P. motoro, P. orbignyi*, and *P. scobina*^52^, in addition to *P. schroederi* and *P. orbignyi*^19^, as well as *P. motoro, P. wallacei* and *P. aiereba*^21^. For the differential leukocyte count, lymphocytes were the predominant cells. Oliveira et al.^20^ and Oliveira et al.^21^ also reported the same results; however, Brito et al.^52^ reported that neutrophils and leukocytes were predominant.

### Cytochemical staining

The existence of neutrophils and eosinophils in the blood of an individual *P. motoro* stingray was reported, and it was difficult to distinguish neutrophils from heterophils^19^. In the present study, no neutrophils were detected. Instead, there were heterophilic granulocytes with morphological features that were distinct from those of neutrophils. However, the granulocytes had heterophilic functions resembling phagocytosis, which is also seen among neutrophils, as indicated by the presence of glycogen, lipids and proteins in *P. wallacei*, *P. motoro* and *P. aiereba*. Glycogen is an important source of cellular energy reserves for the innate defense mechanisms that occur, especially during phagocytosis^37,53^.

In the class Chondrichthyes, the cytochemical characteristics of leukocyte chimeras in the species *Callorhynchus milii* (Bory de Saint-Vincent 1823), *Chimaera phantasma* (Jordan & Snyder 1900), *Hydrolagus novaezealandiae* (Fowler 1911)*, Hydrolagus* sp., *Harriotta raleighana* (Goode & Bean 1895) and *Rhinochimaera pacifica* (Mitsukuri 1895)^54^ were studied. It was reported that the esterase enzyme in the Holocephali subclass was very different from that in elasmobranchs. However, the present study was the first aimed at determining the functions of blood cell types in potamotrygonid species. Positive PAS staining was observed in thrombocytes of *P. wallacei*, *P. motoro* and *P. aiereba*, but the staining in lymphocytes and monocytes was weak. Thrombocytes aid in blood coagulation^55^, but they also play an important role in the immune activity of elasmobranchs^13^.

There was no peroxidase reaction in any of the blood cells of *P. wallacei*, *P. motoro* or *P. aiereba*. Peroxidase is an important lysosomal enzyme involved in intracellular digestion, and one of its main features is that it indicates the absence of eosinophilic and neutrophilic granulocytes in the species investigated here^26^. However, this lack of peroxidase may be accompanied by compensatory development of other antibacterial components, such as cationic proteins^13,38^.

Though basophil leukocytes were rarely observed in the blood of *P. wallacei*, *P. motoro* and *P. aiereba*, their existence was confirmed through metachromasia staining. In addition, the three potamotrygonids demonstrated the presence of lipids in thrombocytes and lymphocytes but to a lesser degree than that in heterophils. Similarly, in *Xiphophorus helleri* (Heckel 1848), a Sudan black reaction was also demonstrated in monocytes and lymphocytes^56^. However, in other teleosts, this reaction has been observed in neutrophil granules^36^. Phagocytic leukocytes can use lipids as an energy source, thereby degrading these constituents through the action of cytoplasmic enzymes.

The proteins in leukocyte granules are involved in host defense and microorganism death^36^. The heterophils and basophils of *P. wallacei*, *P. motoro* and *P. aiereba* were positive for bromophenol blue staining, similar to what had previously been found in eosinophils from *S. brasiliensis*^36^ in Amazonian turtles^26^. Positive staining was observed in basophils, eosinophils and neutrophils from *P. motoro*^19^. Therefore, these results indicate that these proteins play an important role in the innate defense of animals, which is possibly performed by heterophil and basophil granulocytes.

### Ultrastructural analysis

The ultrastructural analyses of leukocytes from *P. wallacei*, *P. motoro* and *P. aiereba* were similar to each other and comparable with the findings from the sharks *G. cirratum*^57^ and *S. canicula*^47^. The morphology and sizes of the different cell types were similar to those of marine rays and sharks. It is very important to characterize the types of stingray leukocytes to provide basic information about these cells and make correlations with health conditions. In this manner, leukocytes can be quantified in these stingrays, which are extremely important for the aquarium industry. The cytochemical characteristics of heterophils indicates that these major granulocytes are important in the immune defense of Amazonian potamotrygonids. The blood cell features of wild native stingrays may be useful for making diagnoses and comparisons among these same species under controlled conditions.

## Data Availability

Data supporting the findings of this manuscript are available from the corresponding author upon reasonable request.
